# Old and Young Sleeping Together

**Published:** 1890-03

**Authors:** 


### OLD AND YOUNG SLEEPING TOGETHER.

A prominent medical writer in discoursing upon this by no means uncommon practice says :

"A habit which is considerably prevalent in almost every family of allowing children to sleep with the older persons, has ruined the nervous vivacity and physical energy of many a promising child. Every parent who loves his child, and wishes to preserve to him a sound nervous system, with which to buffet successfully the cares, sorrows, and labors of life, must see to it that his nervous vitality is not absorbed by some diseased or aged relative.

"Children, compared with adults, are electrically in a positive condition. The rapid changes which are going on in their little bodies abundantly generate and as extensively work up vital nervo-electric fluids. But when, by contact for long nights with elder and negative persons, the vitalizing electricity of their tender organizations is absorbed, they soon pine, grow pale, languid, and dull, while their bed companions feel a corresponding invigoration. It is undeniable that healthful influences are lost, and to a fatal extent sometimes by this ill-advised custom. A woman was prostrated with incurable consumption. Her infant occupied the same bed with her almost constantly day and night. The mother lingered for months on the verge of the grave—her demise being hourly expected. Still she lingered on, daily disproving the predictions of her medical attendants. The child, meanwhile, pined without any apparent disease. Its once fat little cheeks fell away with singular rapidity till every bone in its face was visible. Finally it had imparted to the mother its last spark of vitality and simultaneously both died."

